# Nanosphere Lithography of Chitin and Chitosan with Colloidal and Self-Masking Patterning

**DOI:** 10.3390/polym10020218

**Published:** 2018-02-23

**Authors:** Rakkiyappan Chandran, Kyle Nowlin, Dennis R. LaJeunesse

**Affiliations:** Department of Nanoscience, Joint School of Nanoscience and Nanoengineering, University of North Carolina Greensboro, Greensboro, NC 27401, USA; rakkiyappanchandran@gmail.com (R.C.); ksnowlin@gmail.com (K.N.)

**Keywords:** chitin, chitosan, nanostructured biomaterial, polymer, self-masking nanosphere lithography, cicada

## Abstract

Complex surface topographies control, define, and determine the properties of insect cuticles. In some cases, these nanostructured materials are a direct extension of chitin-based cuticles. The cellular mechanisms that generate these elaborate chitin-based structures are unknown, and involve complicated cellular and biochemical “bottom-up” processes. We demonstrated that a synthetic “top-down” fabrication technique—nanosphere lithography—generates surfaces of chitin or chitosan that mimic the arrangement of nanostructures found on the surface of certain insect wings and eyes. Chitin and chitosan are flexible and biocompatible abundant natural polymers, and are a sustainable resource. The fabrication of nanostructured chitin and chitosan materials enables the development of new biopolymer materials. Finally, we demonstrated that another property of chitin and chitosan—the ability to self-assemble nanosilver particles—enables a novel and powerful new tool for the nanosphere lithographic method: the ability to generate a self-masking thin film. The scalability of the nanosphere lithographic technique is a major limitation; however, the silver nanoparticle self-masking enables a one-step thin-film cast or masking process, which can be used to generate nanostructured surfaces over a wide range of surfaces and areas.

## 1. Introduction

The surfaces of many insects are decorated with nanostructure topographies that control and determine specific physical and chemical properties. The wings and eyes of many insects—including cicadas and moths—have arrays of multimodal nanoscale cones and cylinders that are anti-reflective, anti-wetting, self-cleaning, and anti-microbial [1,2,3,4,5]. Insect cuticles are complex natural composite materials that are composed of a polysaccharide chitin fiber network and a matrix of proteins and lipids, and can potentially form through complex interactions during cuticle deposition [6,7]. While many of these nanostructures in insect cuticles are surface features composed of a lipid–protein matrix, in the case of the dog day cicada (*Tibicens* species), the nanostructured surface is, in part, controlled by deeper extensions of the underlying chitin exoskeleton. This demonstrates that, in some cases, the nanoscale organization of chitin assists in the formation of nanoscale topology [4]. Many insect nanostructured surfaces demonstrate a hexagonally close-packed array nanostructure [4,6,8]. While the mechanisms that control this type of nanoscale chitin organization are unresolved, in this paper, we take a direct approach to organize chitin and the related polysaccharide chitosan at the nanoscale, using a technique called nanosphere lithography (NSL), which utilizes an hexagonal close packed (HCP) patterning method [9,10,11,12,13]. NSL is a two-step fabrication technique that enables the generation of micro- and nanoscale topographies that are composed of hexagonally close-packed arrays of cylindrical, conical, or hemispherical structures [9,10,11,12,13]. The initial step of the NSL process involves the masking of a substrate with an HCP monolayer nanosphere film, which is followed by the processing of the masked substrate using either standard deposition (e.g., plasma vapor deposition), or etching techniques (e.g., reactive ion etching). NSL has been used to generate micro- and nanostructured surfaces in hard semiconductor and synthetic polymeric materials [12,13]. In this work, we demonstrate the generation of a nanostructured surface in a biopolymer—in this case, either the chitin-mats derived from an insect wing, or a thin film composed of chitosan. The nanostructured surface (NSS) generated by NSL mimics the surfaces of cicada wings, however, these synthetic biopolymer surfaces are dynamic and change their morphology in an aqueous environment. One of the major issues with NSL as a large-scale processing technique is its scalability. This limitation is largely due to the difficulty of generating large areas of the nanosphere mask [9,10]. In this paper, we utilize a property of chitin and chitosan—the ability of these polymers to generate silver nanoparticles (AgNP) [14]—as a means to bypass the application of the nanosphere of the substrate in the traditional NSL process. Using a drop-cast chitosan/AgNP as a self-masking thin-film etching substrate, we generated a nanostructured AgNP/chitosan surface. This self-masking technique has great application potential for the large-scale fabrication of nanostructured polymeric surfaces, especially for large and non-uniform areas. 

## 2. Methods

**Chitin and chitosan sources:** For these experiments, chitin was prepared from insect wing cuticles from the periodic 17-year cicada Brood II, *Magicicada septendecim*, collected locally in Greensboro, North Carolina, during the June 2013 emergence. The chitin from these wings was prepared as previously described [4]. Chitosan (molecular weight: 150,000, 1.5% *w*/*v*), acetic acid, NaOH, and NaCl were purchased from Sigma-Aldrich (St. Louis, MO, USA).

**Synthesis of silver nanoparticle in chitosan solution:** AgNO_3_ (>99%) was purchased from Sigma Aldrich chemicals, and was prepared as a 10^−2^ M solution. Chitosan (0.5 g, dissolved in 10 mL of 1% *v*/*v* acetic acid solution) and 0.2 M NaCl in a 5 mL solution were added dropwise. Mixtures of chitosan and AgNO_3_ solution were prepared in a 1:5 ratio (by volume). For the formation of monodispersed nanoparticles, the mixed sample solutions underwent ultra-sonication for 3–4 h. The samples were then drop-casted and dried in oven for about 10 min at 40 °C to make films, and were then assessed for various analytical characteristics.

**Colloidal/nanosphere lithography**: We fabricated 2D HCP monolayer nanosphere (NS) crystals composed of four polystyrene NSs using an indirect method, via assembly at an air-liquid interface, as previously described [13]. We etched the NS-masked and self-masking chitin and chitosan substrates using the South Bay Technology Model PC-2000 Plasma Cleaners (South Bay Technology, San Clemente, CA, USA), with oxygen as the process gas. We performed a 90 s reactive ion etching (RIE) of the surface using the following process parameters: 100 W forward power, −700 V DC bias, and 200 mT chamber pressures.

**SEM imaging:** Scanning electron micrographs were obtained using a Zeiss Auriga FIB/SEM (Carl Zeiss Microscopy, LLC, Thornwood, NY, USA) with accelerating voltage 2–4 kV (30 μm or 7.5 μm aperture), and an Inlens SESI detector. Samples were sputter-coated with an approximately 5 nm gold layer using a Leica EM ACE200 (Leica Microsystems, Buffalo Grove, IL, USA), with real-time thickness monitoring using a quartz crystal microbalance. 

## 3. Results

**Figure 1:**
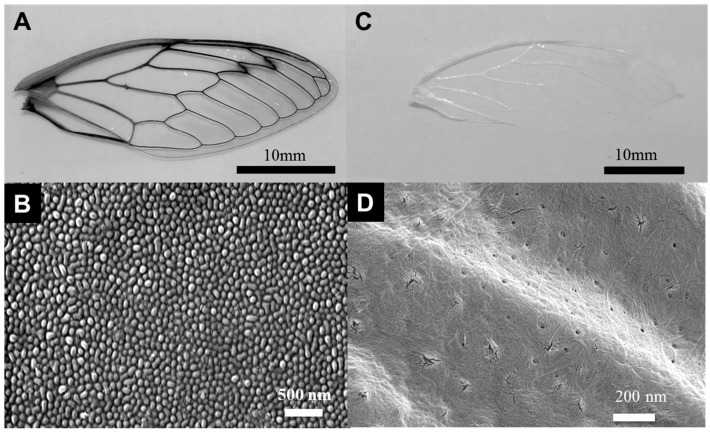
The source of chitin for the colloidal/nanosphere lithography substrate. (**A**) A forewing from the periodic 17-year cicada Brood II *Magicicada septendecim*; (**B**) SEM micrograph of the inter-vein (clear) regions of a wing showing an array of hemispherical nano-features, which is a rough hexagonal close-packed arrangement; (**C**) A forewing from the periodic 17-year cicada Brood II *Magicicada septendecim* after in situ chitin purification. Note the loss of all pigment and color from the wing; (**D**) The surface of the inter-vein region showing the loss of the array of nanofeatures, and the presence of a nanoscale fibrous network.

**Figure 2:**
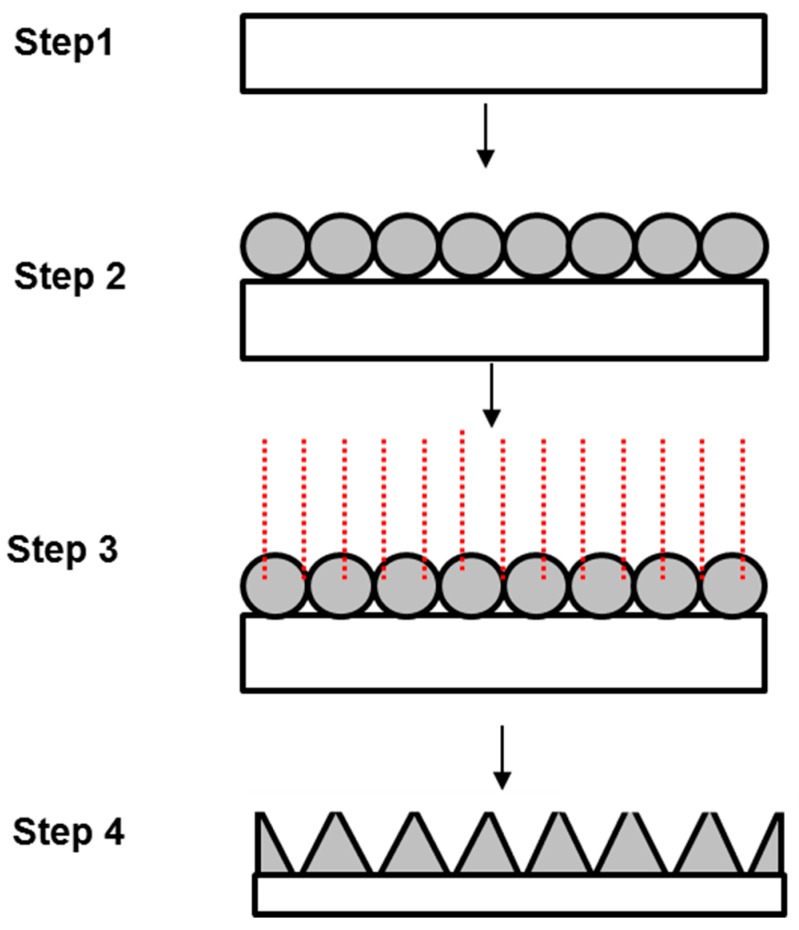
Schematic of the colloidal/nanosphere lithographic process. Step (1) preparation of biopolymer substrate; Step (2) nanosphere masking of substrate; Step (3) Reactive ion etching of masked substrate; and Step (4) completed NSS.

To determine whether a purified chitin scaffold could serve as a substrate for colloidal/nanosphere lithography, we prepared a purified chitin scaffold from a wing of the periodic 17-year cicada Brood II *Magicicada septendecim* (Figure 1A). The wing of the Brood II cicada is decorated by a low-aspect ratio nanostructured surface with a hexagonal close-packed arrangement (Figure 1C) [5]. Previous work has demonstrated that these nanoscale hemispherical structures are predominately protein or wax in composition, and do not retain any nanostructures after the in situ chitin purification procedure [4]. The in situ-purified Brood II cicada wing retained the general appearance of a cicada wing, but lacked any pigmentation or nanostructure (Figure 1C,D). This purified surface was then masked with a 300 nm polystyrene nanosphere monolayer as previously described [13], and used as the etching target for NSL (Figure 2). 

**Figure 3:**
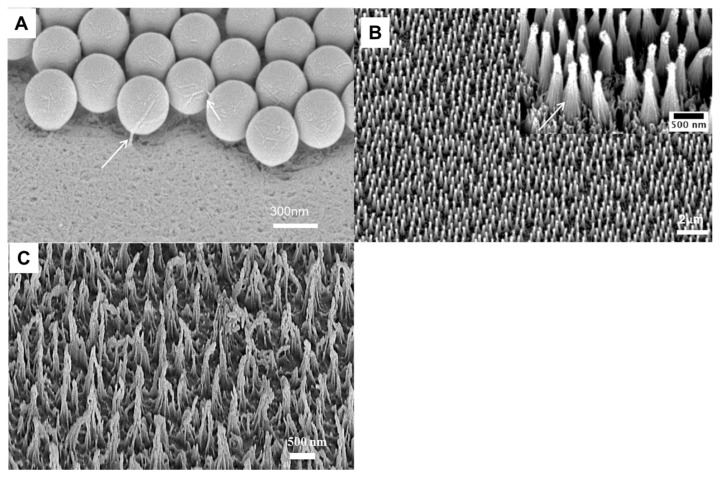
Colloidal/nanosphere lithography on chitin substrate derived from the wing of the periodic 17-year cicada Brood II *Magicicada septendecim.* (**A**) A nanosphere mask on the chitin substrate. Notice the filaments emanating from the chitin surface to the nanospheres, and the presence of a fibrous network on the nanospheres (as noted by the arrows); (**B**) An array of chitin nanocones after the etching process. The inset shows the presence of longitudinal ridges along the nanocones; (**C**) The array of chitin nanocones after incubation in an aqueous solution; note the loss of organization in the array and the structure of the nanocones.

Unlike previous masking substrates, both polymeric and semiconductor [9,13], chitin nanofibers interacted with the polystyrene nanospheres within the mask, showing both projections from the surface and the nanofiber coating of the spheres themselves (Figure 3A, arrows). Chitin substrates prepared from the wings of cicadas have a random organization of nanofibers [4]. Although we do not know what controls this type of interaction (chitin has a low solubility in aqueous solutions [15] and the masking step is performed within an aqueous bath [13]), we suspect that the polystyrene beads are redepositing chitin from the substrate onto the surface. A 90 s reactive ion etch of the nanosphere-masked chitin substrate resulted in a nanostructure surface dominated by high aspect ratio nanocones arranged in a hexagonal close-packed arrangement (Figure 3B). 

The surface of the chitin derived from the wing of the periodic 17-year cicada Brood II *Magicicada septendecim* now resemble the wings of cicadas that have surfaces with higher-aspect ratio nanofeatures [4,5,16]. Closer inspection of these chitin nanocones shows these structures—akin to the previously-described synthetic polymer structures—have secondary finer nanoscale structures (longitudinal ridges) along the length of the nanocones (Figure 3B, inset arrows). We suspect that like the synthetic polymers, these longitudinal nano-ridges are a product of the interaction of the reactive ions and nanoscale structure within the chitin fiber [14]. Chitin nanofibers in natural systems range in size from 2 nm to 10 nm, depending on the context [4,15,17]. In synthetic systems, chitin nanofibers have been demonstrated to self-assemble into nanoscale fibers ranging from 5 nm to 20 nm, depending on solvent conditions [18,19]; the filamentous ridges fall within these ranges. Unlike the nanostructured surfaces generated from synthetic polymers, chitin nanostructured surfaces are extremely labile in aqueous solutions, and unravel to form random tangles of chitin nanofibers (Figure 3C). The dynamic nature of chitin nanocones may serve in applications like drug delivery, in which a dynamic surface with nanoscale features could allow for response to a changing environment. 

**Figure 4:**
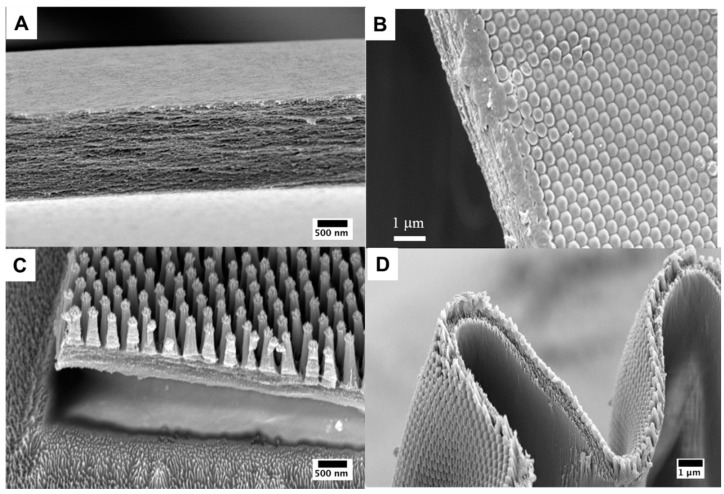
Colloidal/nanosphere lithography on drop-cast chitosan thin films. (**A**) A drop-cast chitosan thin film; note the layers of chitosan nanoscale fibers; (**B**) 300 nm nanosphere mask on chitosan thin film—note the distortion of the polystyrene nanospheres as they are stretched across the surface of the chitosan substrate; (**C**) Post-reactive ion etch of a nanosphere-masked chitosan substrate—note the high-aspect ratio nanocone arrays; (**D**) Flexible nanostructured chitosan thin film.

We also performed colloidal/nanosphere lithography on drop-cast thin films of chitosan. Drop-cast chitosan thin films (~1 µm thick) demonstrated a finer organization than chitin (Figure 4A) thin film, and displayed a stronger interaction with the polystyrene nanospheres, literally stretching or distorting the nanospheres across its surface (Figure 4B). Post-etching, the chitosan substrates displayed a similar arrangement of nanocones as the chitin surfaces (Figure 4C); the nanostructured chitosan thin films were flexible and could be contorted post-etch (Figure 4D). 

**Figure 5:**
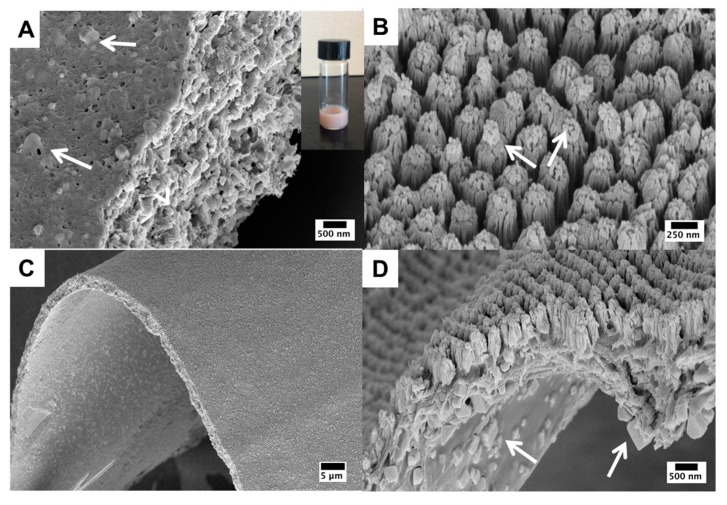
Nanostructured surfaces generated on a self-masking AgNP–chitosan thin film by colloidal-nanosphere lithography. (**A**) Chitosan–AgNP composite thin film generated by a chitosan–AgNP solution (inset); note the presence of cuboidal AgNP embedded within the film (denoted by arrows) (**B**) Post-reactive ion etch of the self-masked chitosan–AgNP composite thin film; note that the array is less-organized without a nanosphere mask compared to with one, and note the presence of colloidal AgNP at the nanocone apex. (**C**,**D**) Demonstration of the flexibility of the nanostructured chitosan–AgNP composite thin films.

Due to the presence of stabilizing and reducing components within their composition, chitin and chitosan have been used for the generation of silver nanoparticles (AgNP) [14]. One of the scaling limitations of colloidal/nanosphere lithography is the masking step. Typically, this step involves either the self-assembly of a nanosphere monolayer at a gas–liquid interface, or the mechanical generation of a nanosphere monolayer via centrifugation [9]; in either case, the area masked is limited. We hypothesized that a substrate with spatially distributed components and inherent differential etching rates would enable a simplified colloidal/nanosphere lithographic procedure. We tested this hypothesis by generating a composite chitosan–silver nanoparticle thin film (Figure 5A). To do this, we used a chitosan solution doped with silver nitrate; this solution generated cuboidal silver nanoparticles and acquired a characteristic purple hue (Figure 5B). We characterized the presence and size of the silver nanoparticles in the chitosan solution using EDX, DLS, and UV-Vis spectroscopy. SEM imaging of the dried thin film produced by drop-casting revealed the presence of fairly evenly-distributed cuboidal AgNP, between 40–60 nm (Figure 5A, arrows). A 90 s reactive ion etch of the chitosan–AgNP composite thin film resulted in a nanostructured surface (Figure 5B). This nanostructured surface was similar in appearance to those generated using a polystyrene nanosphere mask, although the self-masked AgNP-nanostructured surfaces demonstrated far less organization compared to the neatly-arranged hexagonally close-packed nanocones generated from the nanosphere-masked surface, as expected. The silver nanoparticles accumulated on the peaks of the rough chitin nanocones (Figure 5B). As with nanostructured chitosan surfaces, these self-masked chitosan surfaces were flexible (Figure 5C,D), and thus this procedure or modifications of this procedure can be used to coat a variety of materials that are in mechanically dynamic environments (e.g., cell scaffolds, tissue implants). 

## 4. Discussion

Nanostructured surfaces are a powerful means of controlling and changing the surface properties of a material [20,21]. Naturally-structured chitin in insects and in other invertebrates has been demonstrated to have compelling physical properties [4,7]. Among the most interesting are the silica–chitin composite spicules of the glass sponge, *Sericolophus hawaiicus*, which have been shown to be the first biomaterial to display supercontinuum generation [22]. While much of the previous efforts in colloidal/nanosphere lithography has been focused on the modification or application of surface topography to semiconductor materials (and most recently synthetic polymers) [9,13], no work has been done using biopolymers. Chitin and chitosan have long been used as a template for nanoparticle synthesis and the deposition of nanoscale materials [23,24,25,26,27]. Many of the chitin–metal oxide nanocomposite materials are generated by a hydrothermal process, and display enhanced nanoscale topography that synergizes with compositional contributions to the properties and the functionality of these new and interesting materials [23,25]. However, little work has been done to directly structure chitin using standard etching techniques. This work demonstrates that biopolymers like chitin and chitosan are not only compatible with the nanosphere lithographic process, but also provide unique and powerful opportunities for this fabrication technique—especially for the generation of antimicrobial surfaces and biomimetic optical materials. The chemical and structural properties of chitin and chitosan (e.g., their nanoscale fiber size and organization) enables the generation of dynamic and responsive nanostructure surfaces which can be used to release drugs or growth factors in response to changes in the local environment. The self-assembly of metallic nanoparticles by chitin and chitosan has enabled the generation of a self-masking substrate for a colloidal/nanosphere lithographic process. While further work is needed to refine the self-assembly of the metallic nanomaterials and their distribution within the chitosan thin film, using chitosan or other polymers enables the large-scale application of nanostructure surfaces to a broad range of targets via a colloidal/nanosphere lithographic-like approach.
